# A computational method for clinically relevant cancer stratification and driver mutation module discovery using personal genomics profiles

**DOI:** 10.1186/1471-2164-16-S7-S6

**Published:** 2015-06-11

**Authors:** Lin Wang, Fuhai Li, Jianting Sheng, Stephen TC Wong

**Affiliations:** 1 NCI Center for Modeling Cancer Development, Department of Systems Medicine and Bioengineering, Houston Methodist Research Institute, Weill Cornell Medical College, Houston, Texas 77030, USA

## Abstract

**Background:**

Personalized genomics instability, e.g., somatic mutations, is believed to contribute to the heterogeneous drug responses in patient cohorts. However, it is difficult to discover personalized driver mutations that are predictive of drug sensitivity owing to diverse and complex mutations of individual patients. To circumvent this problem, a novel computational method is presented to discover potential drug sensitivity relevant cancer subtypes and identify driver mutation modules of individual subtypes by coupling differentially expressed genes (DEGs) based subtyping analysis with the driver mutation network analysis.

**Results:**

The proposed method was applied to breast cancer and lung cancer samples available from The Cancer Genome Atlas (TCGA). Cancer subtypes were uncovered with significantly different survival rates, and more interestingly, distinct driver mutation modules were also discovered among different subtypes, indicating the potential mechanism of heterogeneous drug sensitivity.

**Conclusions:**

The research findings can be used to help guide the repurposing of known drugs and their combinations in order to target these dysfunctional modules and their downstream signaling effectively for achieving personalized or precision medicine treatment.

## Background

It is a long-standing problem in cancer treatment that drugs often have heterogeneous responses and show sensitivity in subsets of patient cohorts [1,2]. The diverse genomics instability is believed to be responsible for the heterogeneity of drug response [3]. Two large-scale datasets, i.e., the Cancer Cell Line Encyclopedia (CCLE) [4] and Genomics of Drug Sensitivity in Cancer (GDSC) [5], have recently been released independently to study the causal relationship between hundreds of drugs' sensitivity and genomics aberrations of 1,000 cancer cell lines. On the other hand, The Cancer Genome Atlas (TCGA) [6] project profiles the genomics of about 10,000 patient samples across over 30 cancer types. The integrative analysis indicated multiple subtypes of cancers with complex genomics characteristics, e.g., breast cancer [7], squamous cell lung cancer [8,9], and Uterine cancer [10]. Usually, mRNA data from RNA sequencing and microarrays [8,9] and somatic copy number alterations (SCNAs) or genetic mutation were used to conduct the subtyping analysis by comparing the difference among cancer samples [10]. However, subtyping analyses often have complex genomics signatures, and thus failed to offer insight into drug sensitivity and to identify the predictive driver mutations.

In this paper, we present a new integrative approach to circumvent this problem by dividing cancer patients into clinically relevant subtypes based on comparing differentially expressed genes (DEGs) with the normal (rather than comparing among cancer samples) and uncover driver mutation modules (rather than individual mutations) of individual subtypes based on the network analysis. Our hypotheses are that: 1) distinct and mutual mutations in a network module can cause the same dysfunctional signaling pathways, and 2) the dysfunctional signaling pathways are indicated by DEGs. The mutual mutation module is necessary for cancer and taken advantage of the low frequency of individual mutations [11]. Another advantage of using the ranking of DEGs is that the subtypes sharing the common DEGs also have higher possibility sharing the effective drugs. Drug repositioning [12,13], drug combinations [14,15] and mechanism of action (MoA) delineation [16] based on reverse differential gene expression profiling are becoming popular and important drug discovery studies, and are accelerated means to find new indications of existing drugs [17,18].

## Results

### Methodology overview

**Figure 1:**
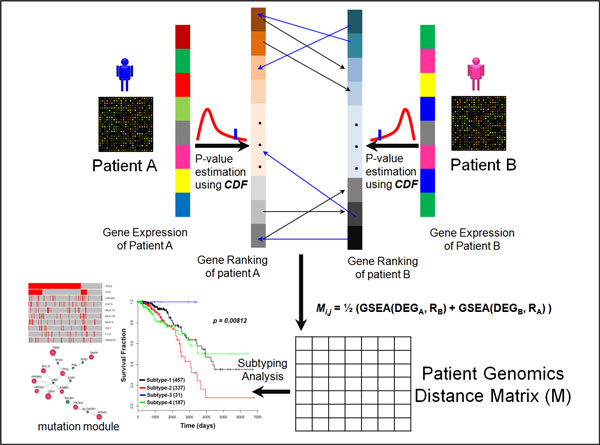
**Methodology Overview**. The cumulative distribution function (CDF) of individual genes in the tumor tissue is calculated in a fitted Gaussian, and the mean and variance are estimated from the pooling of normal samples. Then the genes of individual cancer patients are ranked based on their p-values that indicate how far their expression values are away from the normal. The 200 differentially expressed genes (DEGs) are selected based on their ranking of p-values (smaller ones). The distance (difference) between any two patients is calculated by using the average gene set enrichment analysis (GSEA) scores of the DEGs of the two patients. Consequently, the subtyping analysis is applied on the sample distance matrix to discover the drug response subtypes and mutation modules.

Figure 1 shows the overview of the methodology. In specific, the mRNA expression data of individual cancer patients are downloaded from TCGA. To identify the differentially expressed genes for each patient, the expressions of individual genes in the normal tissue are fitted by a Gaussian distribution with the mean and variance estimated from the pooling of normal samples. The p-value of each gene, which indicates how far the expression of the gene in the cancer sample is away from the normal, is obtained by calculating the cumulative distribution function (CDF) of observing the given gene expression level, or higher for the up-regulated genes and lower for the down-regulated genes. The top 100 up-regulated and 100 down-regulated genes are selected as the set of differentially expressed genes (DEGs) for the calculating of gene set enrichment analysis (GSEA) score [19]. The selected set of DGEs and gene ranking of any two patients are used as inputs of GSEA to calculate the distance between them. Subtyping (clustering) analysis is conducted based on the sample distance matrix to obtain the drug response relevant subtypes. Finally, the mutation data are extracted for each subtype and linked into the driver mutation module through network analysis.

### Breast cancer stratification

**Figure 2:**
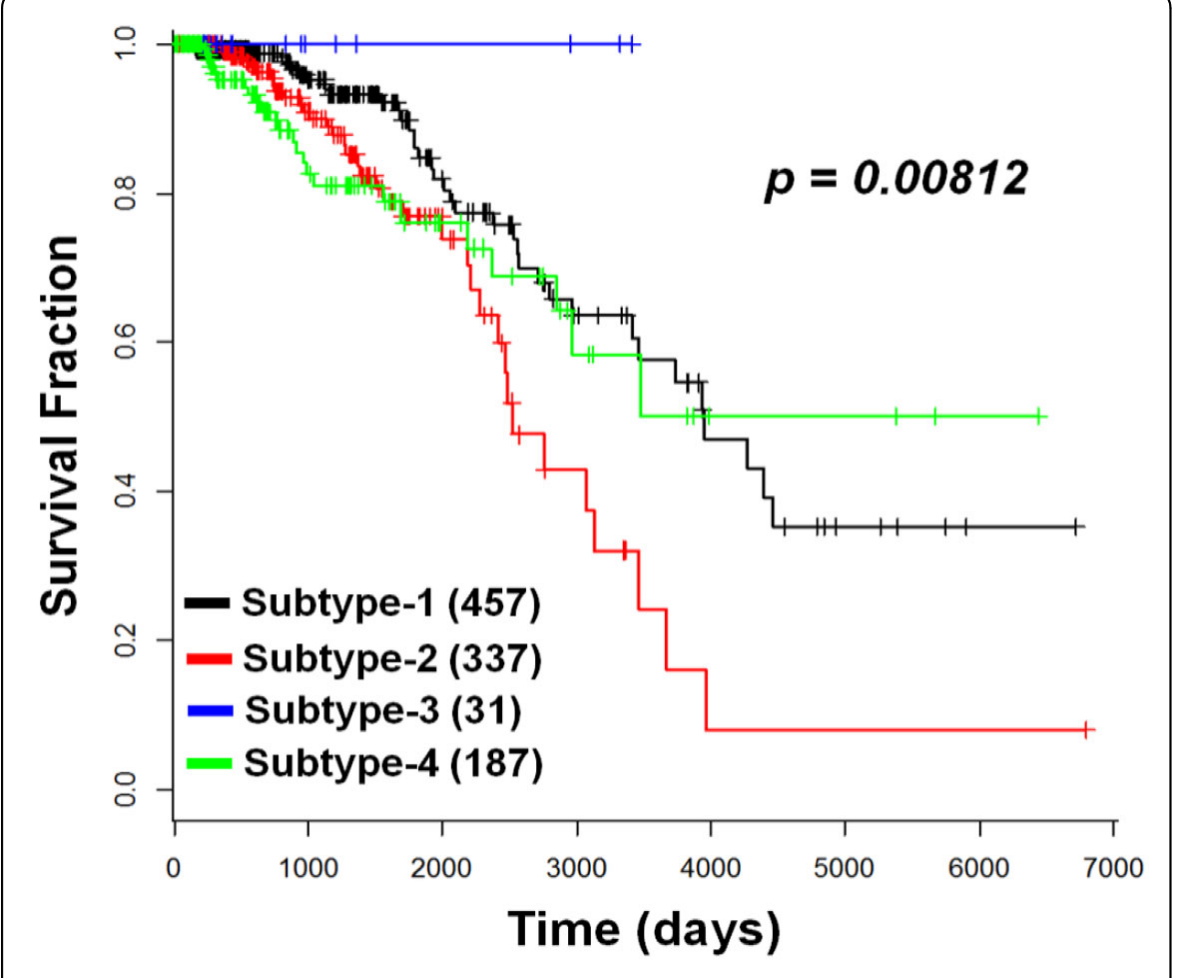
**Survival analysis of breast cancer stratification**. Four subtypes were obtained with the number of patients in each cohort: 457, 337, 31 and 187, respectively. Four subtypes of BRCA were obtained with 457, 337, 31, and 187 patients respectively. The p-value of the significant difference of survival among the four subtypes is 0.00812. Subtype 1: black curve; subtype 2: red curve; subtype 3: blue curve; subtype 4: green curve

1,012 breast cancer patients' and 111 normal subjects' mRNA profiles, as well as their corresponding mutation profiles are downloaded. With the stratification strategy, as shown in Figure 2, four subtypes of BRCA were obtained with 457, 337, 31, and 187 patients respectively. The p-value, 0.00812, shows the significant difference of survival among the four subtypes, which partially indicates the clinical relevance of the subtypes. Surprisingly, all patients of subtype 3 (blue curve) survived during the study period (about 4,000 days), whereas the subtype 2 (red curve) had a poor prognosis.

**Figure 3:**
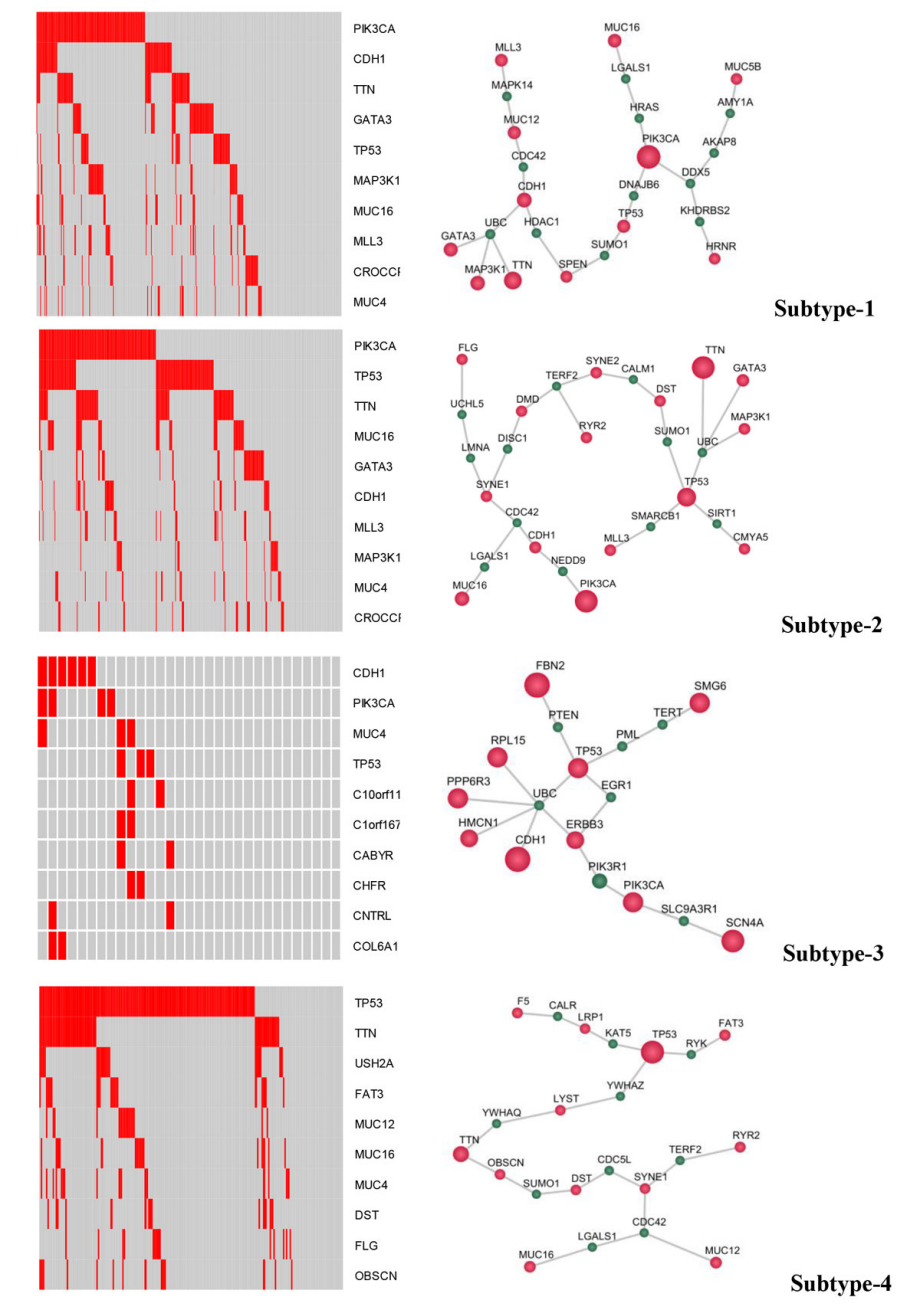
**Driver mutations signatures (left panel) and network modules (right panel) of four breast cancer subtypes**. (Red indicates driver mutations, and cyan means the connection nodes. Left panel: the heat maps of the top ten high-frequency mutated genes of four subtypes. The column label: the patient samples ID of each subtype; the row label: ten high-frequency mutated genes ID. The red color in the heat map represents mutation detected in the corresponding sample, and the gray color indicates that a gene has no mutation in the corresponding sample. Right panel: the networks of top ranked mutations of four subtypes. Red nodes: mutated genes; green nodes: connections genes.

To discover the underlying driver mutations, the mutation data from DNA sequencing was extracted. For each subtype, the top ten high-frequency mutations, which include genes mutated in more than 10% of patients of each subtype, are shown in Figure 3**-left panel**. The column label of the heat map is the patient sample ID, and the red color in the heat map represents mutation detected in the corresponding sample, and the gray color indicates that a gene has no mutation in the corresponding sample. Subtype-3 has only a few mutations and has the best prognosis outcomes. On the other hand, both TP53 and PIK3CA mutations are top ranked in subtype-2, which might explain the poor prognosis. The top ranked mutations are further connected into network modules, as shown in Figure 3**-right panel**. Different genes were involved in the driver mutation network for individual subtypes, and they are linked via one or two connection genes. These driver mutation modules offer insight into the dysfunctional modules that regulate the prognosis and drug responses.

### Adjusted survival analysis of breast cancer

**Figure 4:**
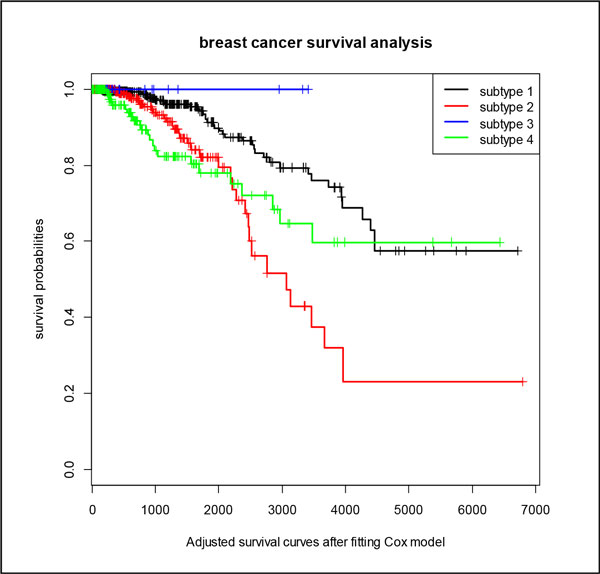
**Adjusted survival curves of breast cancer after fitting Cox model**. The adjusted four survival curves of four breast cancer subtypes after fitting to the Cox model to eliminate the other characteristics that significant impact on the prognosis and survival.

We fitted the survival of breast cancer in COX regression model and found the important characteristics that can affect the prognosis and survival from the clinical information of breast cancer in TCGA. These factors are "menopause status", "margin status", "ajcc nodes pathologic pn", "ajcc pathologic tumor stage" and "age". Figure 4 shows the adjusted survival curves of breast cancer after fitting in the COX model. We can see that the survival curves after adjustment have only minor changes compared with previous survival curves, which means the clustering results are stable.

### Lung cancer stratification

For lung adenocarcinoma (LUAD), 443 cancer patients' and 58 normal subjects' mRNA profiles, and their corresponding mutation profiles are downloaded. With the stratification strategy, five subtypes of LUAD were obtained from five cohorts of 99, 59, 83, 53, and 149 patients respectively based on the best p-value, as shown in Figure 4. The p-value, 0.00251, shows the significant difference of survival among the five subtypes. Though all the LUAD subtypes have relatively poor prognoses (survival rate is about 10%) comparing to breast cancer after 8 years (after 3,000 days), there are distinct survival patterns before 5.5 years (around 2,000 days).

**Figure 5:**
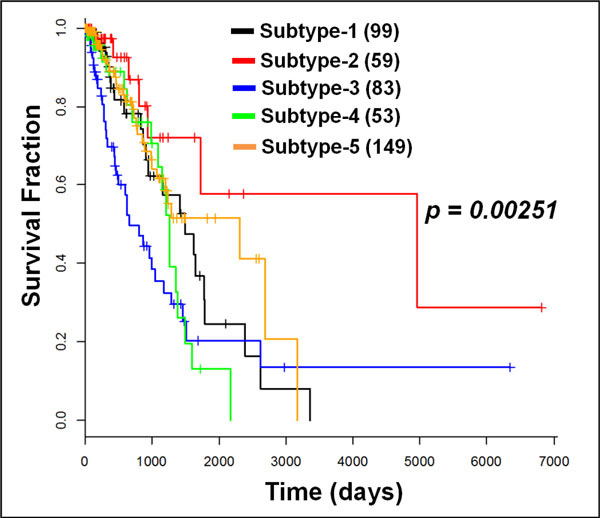
**Survival analysis of lung cancer stratification**. Five subtypes were obtained with 443 numbers of patients: 99, 59, 83, 53 and 149, respectively. Five subtypes of LUAD were obtained with 99, 59, 83, 53 and 149 patients respectively. The p-value of the significant difference of survival among the five subtypes is 0.00251. Subtype 1: black curve; subtype 2: red curve; subtype 3: blue curve; subtype 4: green curve; Subtype 5: brown curve

Figure 5 shows the top twenty high-frequency mutations (the amount of genes that are mutated more than 10% in the patients of each subtype) in the left-panel; the reason may be that LUAD has many more mutations compared with breast cancer. The network modules of the driver mutations are showed in the right-panel. Subtype-2 (red) and subtype-5 (brown) have less TP53 mutation and may contribute to their relatively better prognosis outcomes. On the other hand, the existence of TP53, TTN, MUC16, and CMSD3 mutations in the other three subtypes may be responsible for their poor prognoses. In conclusion, the uncovered driver mutation modules (rather than individual mutations) are important regulators of the clinically relevant subtypes. Therefore, drugs and drug combinations targeting the downstream signaling of TP53 and TTN modules might be effective for better cancer therapy.

### Adjusted survival analysis of lung cancer

**Figure 7:**
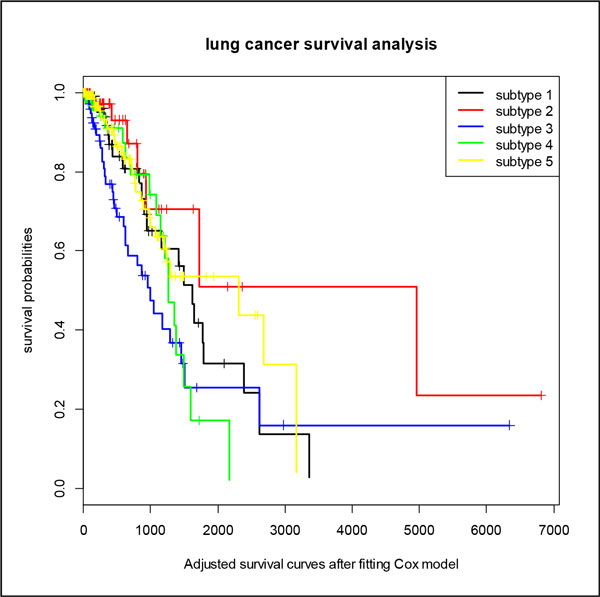
**Adjusted survival curves of lung cancer after fitting Cox model**. The adjusted five survival curves of five lung cancer subtypes after fitting to the Cox model to eliminate the other characteristics that significant impact on the prognosis and survival.

We fitted the survival curves of lung cancer using COX regression model and found the important characteristics that have significant impact on the prognosis and survival from the clinical information of lung cancer in TCGA. We found that, for lung cancer, "the history of other malignancy" and "ajcc pathologic tumor stage" are the important factors. Figure 7 shows the adjusted survival curves of lung cancer after fitting in the COX model. We can see that the survival curves after adjustment also have only small changes compared with original survival curves.

## Discussion and conclusions

Cancer is a complex disease, and individual cancer patients often have heterogeneous genomics instability such that anti-cancer drugs often have profound sensitivity in a subset of patients bearing the same genomic mutation modules. Though large-scale genomics profiling data of individual patients are now available, the genomic mutation modules that regulate drug responses remain mostly unknown. Effective computational methods to mine and extract the knowledge from the large-scale genomics datasets are needed urgently.

Motivated by the successful drug repositioning and discovery of drug mechanism of action by using reverse gene expression profiling, this study presented an integrative computational method to stratify cancer patients into potential clinical correlated subtypes and identify the underlying driver mutation modules that are responsible for the drug sensitivity. The evaluation results on two major types of cancer (breast and lung) reveal subtypes with significant survival time difference and distinct driver mutations of individual subtypes. The proposed method opens up a new paradigm of cancer stratification. More importantly, it uncovers the underlying driver mutation modules (mechanism of drug sensitivity), which might be used to find effective drugs that directly target the mutations or block the downstream signaling of the mutation modules. In future work, we will refine the mutation network modules, and discover synergistic drug combinations blocking the alternative downstream signaling of the mutation network modules, and test the predicted drug combinations on cell lines bearing the same mutation network modules.

## Data and methodology

### Personal genomics data

The mRNA and mutation data from RNA/DNA sequencing, and clinical data (e.g., the survival time information) of breast invasive carcinoma (BRCA), Lung adenocarcinoma (LUAD), as well as the corresponding normal samples, were obtained from TCGA data portal. In total, there are 1,049 mRNA tumor samples (111 normal samples), 1,012 mutation samples of BRCA; 488 mRNA tumor samples (58 normal samples), 443 mutation samples of LUAD.

### Ranking of differentially expressed genes

We use the RNASeqV2 data of breast cancer and lung cancer in TCGA portal. We fitted the individual genes of the normal samples into Gaussian distribution and estimated the normal mean and standard variation by Maximum Likelihood Estimation. Then we calculated the CDF (**C**umulative **D**istribution **F**unction) of the individual genes of each tumor sample in the fitted Gaussian distribution which is the p-value that indicates the significant difference between the tumor sample and the normal samples. The larger value of CDF of one tumor sample gene, the more up-regulated of this tumor sample gene, and vice versa. Then we ranked the genes in each patient based on the p-values, which list the genes from the most up-regulated to the most down-regulated. We called it the ranked gene list for each patient which can be used to calculate the distance between any two patients.

### Calculation of patient genomics distance

The distance between patient A and patient B is determined by the assessment of how similar the two patients' ranked gene lists are. We selected the 100 most up-regulated genes and 100 most down-regulated genes in patient A as the signature, and qualified the distribution of this signature in the ranked gene list of patient B, and vice versa. As for the problem of selecting the best number of up/down regulated genes for the clustering analysis, we tested a series number of up/down genes as 2500, 2000, 1500, 1000, 500, 300, 200, 100, 50 respectively. In general, the clustering results are stable to the number of genes, and then we select 100 up/down regulated genes empirically. We conducted the Gene set Enrichment Analysis (GSEA) [19] using patient A's signature and patient B's ranked gene list, and vice versa as followed. For patient A, we got the enrichment scores of 100 up-regulated genes and 100 down-regulated genes with respect to the ranked gene list of patient B, defined as $ES_{B}^{up}$ and $ES_{B}^{down}$. The Total Enrichment Score (TES) of patient A with respect to patient B is defined as follows:

$$TES_{A,B}=1-\frac{ES_{B}^{up}-ES_{B}^{down}}{2}$$

The $TES_{A,B}$ quantifies the genomics variation difference between patients A and B. Then the distance measurement among patients is defined as: $D_{A,B}=\frac{TES_{A,B}+TES_{B,A}}{2}$, which is the corresponding element in the genomics distance matrix M.

### Clustering analysis

The hierarchical clustering method [20] was employed. After we calculated the distance between every two patients, we got the distance matrix M of N*N, where N is the number of the patient. The distance matrix is used to generate the dissimilarly structure. The cluster method was set as the 'complete linkage' method that tends to find similar clusters.

### Survival analysis

The survival analysis was conducted by using the 'survival' package in R [21]. It uses the cox-proportional hazards model to calculate the patient survival in each subtype using clustering analysis. An associated p-value is calculated to determine the significance of difference among each subtype's survival curve. The patients' 'vital status,' 'last contact day,' and 'death days' were extracted from clinical data of samples downloaded from TCGA.

### Driver mutation module discovery

The PPI data was obtained from BioGRID database [22]. It is a widely used PPI database. For the patients in each subtype of one cancer, we select the top 15 genes based on their mutated frequency and mapped them to the PPI network. An optimal connected sub-network was found for each subtype by solving Steiner tree problem. Let G(V,E) be the background PPI network and $V_{m}$ be the list of top mutated genes. The shortest path for each pair of genes in $V_{m}$ was calculated using Dijkstra's algorithm [23], which is a graph search algorithm that solves the single-source shortest path problem. We use this algorithm to find the shortest path between each pair of the top mutated genes, and then find the minimum spanning tree of the integrated network include all the top mutated genes and their paths as followed. The pairwise distance was calculated and denoted by $D=\left(d_{ij}\right)$. A complete network, $K_{v_{m}}$, was generated with nodes in $V_{m}$. Then $d_{ij}$ was assigned as the edge weight of $\left(v_{i},v_{j}\right)\in E\left(K_{v_{m}}\right)$. The minimum spanning tree of $K_{v_{m}}$, $T_{v_{m}}$, was calculated. We replaced each edge in ${\text{T}}_{{\text{v}}_{\text{m}}}$ with the shortest path between the two end nodes. The genes in the final network were considered as the marker of the subtype.

## Abbreviations

GSEA: Gene set Enrichment Analysis

TES: The Total Enrichment Score

DEG: differentially expressed genes

TCGA: The Cancer Genome Atlas

CCLE: Cancer Cell Line Encyclopedia

GDSC: Genomics of Drug Sensitivity in Cancer

SCNAs: Somatic copy number alterations

MoA: Mechanism of action

CDF: Cumulative distribution function

LUAD: Lung adenocarcinoma

BRCA: Breast invasive carcinoma

## Competing interests

The authors declare that they have no competing interests.

## Authors' contributions

FL, LW, SW designed the experiment, LW, FL, JS, SW wrote the paper. LW, JS, FL carried out all the experiments. All authors read and approved the final manuscript.

**Figure 6:**
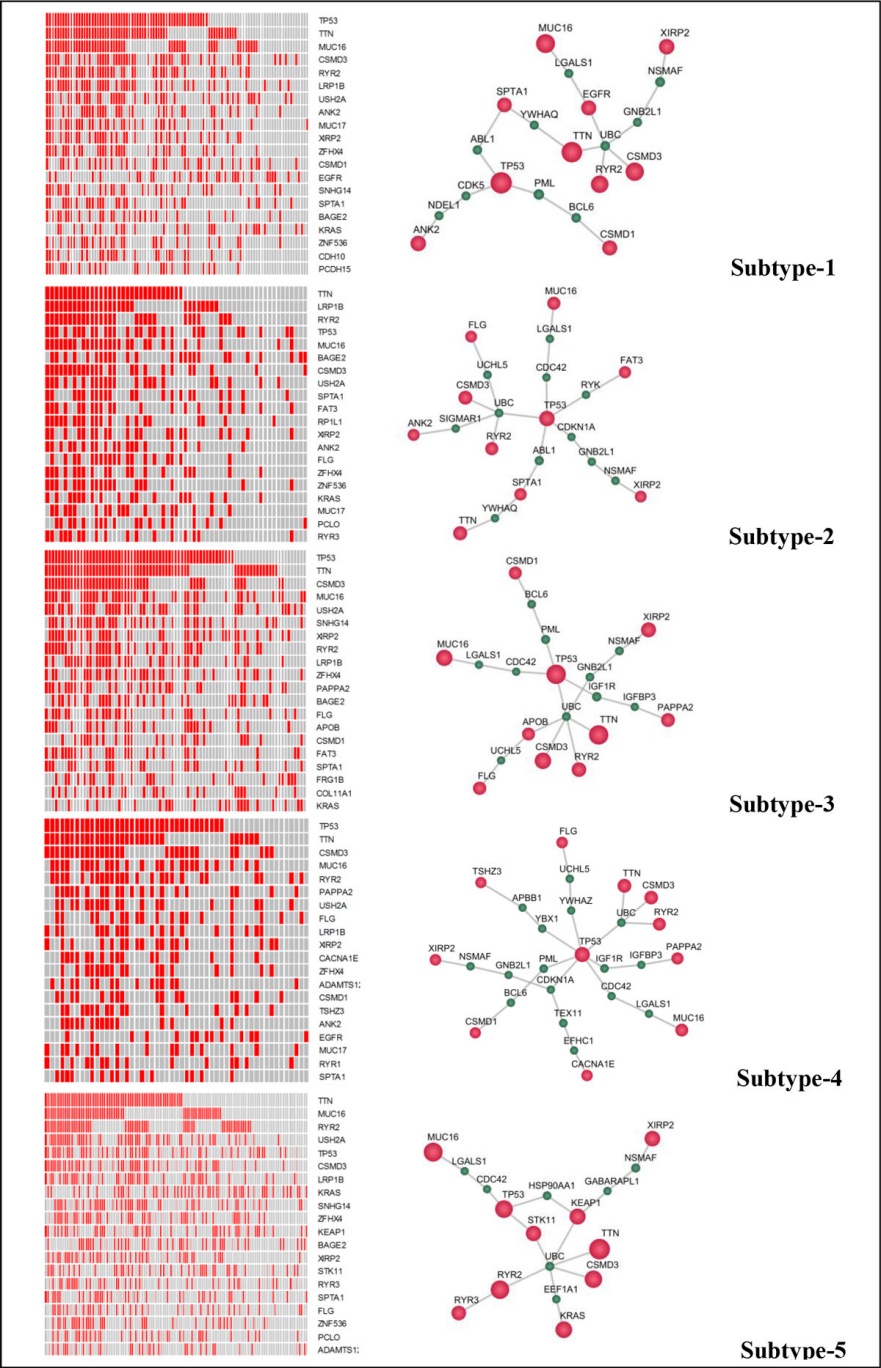
**Driver mutations signatures (left panel) and network modules (right panel) of five lung cancer subtypes**. (Red indicates driver mutations, and cyan means the connection nodes. Left panel: the heat maps of the top twenty high-frequency mutated genes of four subtypes. The column label: the patient samples ID of each subtype; the row label: ten high-frequency mutated genes ID. The red color in the heat map represents mutation detected in the corresponding sample, and the gray color indicates that a gene has no mutation in the corresponding sample. Right panel: the networks of top ranked mutations of five subtypes. Red nodes: mutated genes; green nodes: connections genes.

## Declarations

The publication costs for this article were funded by the corresponding author.

This article has been published as part of *BMC Genomics *Volume 16 Supplement 7, 2015: Selected articles from The International Conference on Intelligent Biology and Medicine (ICIBM) 2014: Genomics. The full contents of the supplement are available online at http://www.biomedcentral.com/bmcgenomics/supplements/16/S7.
